# The vitamin D analogue calcipotriol promotes an anti-tumorigenic phenotype of human pancreatic CAFs but reduces T cell mediated immunity

**DOI:** 10.1038/s41598-020-74368-3

**Published:** 2020-10-15

**Authors:** Laia Gorchs, Sultan Ahmed, Chanté Mayer, Alisa Knauf, Carlos Fernández Moro, Mattias Svensson, Rainer Heuchel, Elena Rangelova, Peter Bergman, Helen Kaipe

**Affiliations:** 1 Department of Laboratory Medicine, Karolinska Institutet, Stockholm, Sweden; 2 Department of Pathology/Cytology, Karolinska University Hospital, Stockholm, Sweden; 3 Department of Medicine, Centre for Infectious Medicine, Karolinska University Hospital, Stockholm, Sweden; 4 Department of CLINTEC, Karolinska Institutet, Stockholm, Sweden; 5 Pancreatic Surgery Unit, Centre for Digestive Diseases, Karolinska University Hospital, Stockholm, Sweden; 6 Infectious Disease Clinic, The Immunodeficiency Unit, Karolinska University Hospital, Stockholm, Sweden; 7 Clinical Immunology and Transfusion Medicine, Karolinska University Hospital, Stockholm, Sweden

**Keywords:** Cancer microenvironment, Adaptive immunity, Cancer, Immunology

## Abstract

The pancreatic tumour stroma is composed of phenotypically heterogenous cancer-associated fibroblasts (CAFs) with both pro- and anti-tumorigenic functions. Here, we studied the impact of calcipotriol, a vitamin D_3_ analogue, on the activation of human pancreatic CAFs and T cells using 2- and 3-dimensional (2D, 3D) cell culture models. We found that calcipotriol decreased CAF proliferation and migration and reduced the release of the pro-tumorigenic factors prostaglandin E_2_, IL-6, periostin, and leukemia inhibitory factor. However, calcipotriol promoted PD-L1 upregulation, which could influence T cell mediated tumour immune surveillance. Calcipotriol reduced T cell proliferation and production of IFN-γ, granzyme B and IL-17, but increased IL-10 secretion. These effects were even more profound in the presence of CAFs in 2D cultures and in the presence of CAFs and pancreatic tumour cell line (PANC-1) spheroids in 3D cultures. Functional assays on tumour infiltrating lymphocytes also showed a reduction in T cell activation by calcipotriol. This suggests that calcipotriol reduces the tumour supportive activity of CAFs but at the same time reduces T cell effector functions, which could compromise the patients’ tumour immune surveillance. Thus, vitamin D_3_ analogues appear to have dual functions in the context of pancreatic cancer, which could have important clinical implications.

## Introduction

Due to the lack of an effective treatment for pancreatic cancer, the five-year survival rate has only increased from 3 to 9% since the mid-1970s^1^. By 2030, it is estimated to become the second highest cause of death in cancer^2^. The main feature of pancreatic ductal adenocarcinoma (PDAC) is that the tumour microenvironment consists of an abundant desmoplastic reaction promoted by resident pancreatic stellate cells and other infiltrating cancer-associated fibroblasts (CAFs)^3^. In the presence of cancer cells, pancreatic stellate cells become activated, lose their characteristic cytoplasmic lipid droplets, express the myofibroblast protein α-smooth muscle actin (α-SMA), and acquire a proliferative phenotype leading to a pathological release of extracellular matrix proteins^4^. The desmoplastic stroma encapsulates the cancer cells, which prevents therapy delivery^3,4^ as well as an efficient effector immune response^5,6^.

Attempts have been made to eradicate CAFs from tumours. However, local depletion of α-SMA^+^ CAFs in experimental PDAC has been shown to be associated with tumour progression^7^. CAFs can act positively or negatively on tumour cell growth based on the expression pattern of factors such as α-SMA^8^, IL-6^8^, leukemia inhibitory factor (LIF)^9^, podoplanin^10,11^ and periostin^11,12^ among others. The existence of different subsets of CAFs with diverse and even opposite functions might explain why depletion of the pancreatic tumour stroma has been shown to result in an increased tumour aggressiveness^13,14^ and poor immune-surveillance response^7^ in murine models and in subsequent clinical trials. Öhlund et al*.* recently identified two phenotypically distinct CAF subsets within pancreatic tumours. α-SMA^+^ myofibroblastic CAFs were localized in close proximity to the tumour nests and were described to restrain tumour cell growth, whereas IL-6 producing inflammatory CAFs were positioned more distantly from the cancer cells and were suggested to be more pro-tumourigenic^8^.

To overcome the difficulty of targeting the tumour-stromal interactions of only the pro-tumorigenic CAF subsets, emerging approaches aim to restore the activated CAFs to a quiescent state, rather than its depletion^15,16^. Sherman et al.^15^ showed that the activation of vitamin D receptor (VDR) by vitamin D_3_ analogues in CAFs results in a reduction of pancreatic fibrosis and an improved response to chemotherapeutic drugs in a murine model of PDAC. However, the immunosuppressive role of vitamin D_3_ and the downstream interactions between immune cells, cancer cells and the stroma in this setting remain elusive.

An effective T cell mediated anti-tumour immunity involves both CD8^+^ T cells and CD4^+^ Th1 subsets. Vitamin D_3_ have previously been shown to promote Th2 immune responses and inhibit Th1 cytokines^17^. Due to the immunosuppressive activity of vitamin D_3_, it has been suggested to be used as an adjuvant for transplant patients^18^ and for patients with autoimmune diseases, such as psoriasis^19^. Controversially, vitamin D_3_ supplements have also been suggested to benefit cancer patients and clinical trials with vitamin D_3_ analogues combined with other standard treatments are currently ongoing (*ClinicalTrials.gov* identifiers NCT02052492, NCT03331562).

However, three independent observational studies have shown divergent results between levels of systemic vitamin D_3_ (25OHD_3_) and risk of developing pancreatic cancer^20–22^. Stolzeberg-Solomon et al*.*^20^ found that high serum levels of vitamin D_3_ was associated with a high risk of developing pancreatic cancer. In contrast, other studies have found that high serum levels of 25OHD_3_ were associated with a lower risk of pancreatic cancer^21^ and prolonged survival^22^. Combined, these results underscore that the role of vitamin D_3_ in pancreatic cancer is still inconclusive.

We have previously shown that pancreatic CAFs have a strong immunosuppressive effect on T cells and promote the expression of co-inhibitory markers, including PD-1, TIM-3, and CTLA-4^23^. We and others have also shown that the majority of T cells are entrapped in the stromal compartment of the tumour, with limited access to the malignant cells, highlighting the importance of increasing the knowledge in how the stromal microenvironment affect T cell responses^5,6,23^. Notably, the role of vitamin D_3_ in this context is not clear. Therefore, we set out to examine how vitamin D_3_ could affect human pancreatic CAFs and its concomitant effects on T cell function. To that end, we performed a series of in vitro assays to investigate the impact of the vitamin D_3_ analogue, calcipotriol, on the activation of human derived CAFs in both two-dimensional (2D) and three-dimensional (3D) models and the possible role of calcipotriol in the regulation of CAFs and T cells.

## Results

### Effects of calcipotriol on CAF activation

**Figure 1:**
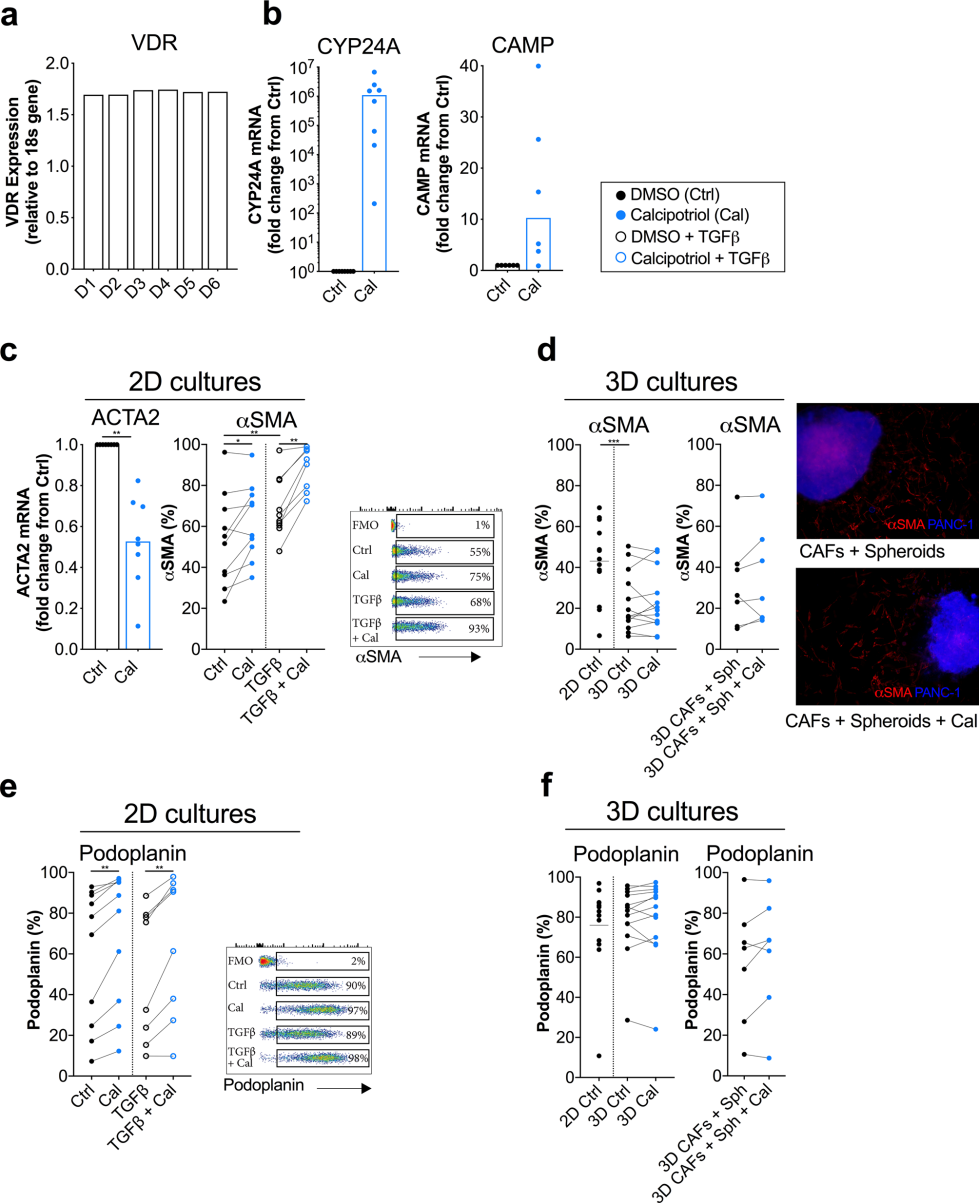
CAFs respond to the vitamin D_3_ analogue calcipotriol. CAFs were treated with either DMSO control (filled circle) or 100 nM calcipotriol (blue filled circle) for 72 h. (**a**) Relative expression levels of VDR gene normalized to the housekeeping gene 18 s in non-treated CAFs (*n* = 6). (**b**) Fold change expression of (Left) CYP24A (*n* = 8) and (Right) CAMP genes (*n* = 6) upon calcipotriol treatment normalized to the DMSO control. (**c**) (Left) fold change gene expression of ACTA2 normalized to the DMSO control (*n* = 8). (Middle) expression of αSMA in CAFs treated with either DMSO control, calcipotriol, (*n* = 11) TGFβ (open circle) or TGFβ together with calcipotriol (blue open circle) (*n* = 8) in 2D cultures. (Right) representative flow cytometry dot plots of αSMA expression with different treatments is shown. (**d**) (Left) expression of α-SMA in CAFs cultured in 2D or 3D models treated with either DMSO control or calcipotriol (*n* = 13). (Middle) expression of αSMA on CAFs co-cultured with spheroids in 3D models (*n* = 7). (Right) immunostaining of the 3D models showing αSMA expression in CAFs (in red) co-cultured with PANC-1 spheroids (in blue) is shown. (**e**) Expression of podoplanin on CAFs treated with either DMSO control, calcipotriol (*n* = 10), TGFβ or TGFβ together with calcipotriol (*n* = 8) in 2D cultures. Representative flow cytometry dot plots of podoplanin expression with different treatments is shown. (**f**) (Left) expression of podoplanin in CAFs cultured in 2D or 3D models treated with either DMSO control or calcipotriol (*n* = 13). (Right) expression of podoplanin on CAFs co-cultured with spheroids in 3D models (*n* = 7). (**a**–**c**) Bars of histograms represent the median. (**c**–**f**) Lines between dots indicate paired samples. Wilcoxon matched-pairs singed rank test was used to detect statistically significant differences *p < 0.05, **p < 0.01, ***p < 0.001.

Primary cultures of CAFs were established from human pancreatic tumour tissues derived from pancreatic cancer patients undergoing surgical resection. We first examined the expression of the vitamin D receptor (VDR), and in line with the results of others^15^, the VDR was expressed in the isolated CAFs (Fig. 1a). To evaluate whether the CAFs were able to respond to the synthetic vitamin D_3_ derivate calcipotriol, we measured the relative mRNA expression of the VDR target gene *CYP24A1* in response to the vitamin D_3_ analogue. The expression of the *CYP24A1* gene (n = 8) was upregulated in all donors after addition of calcipotriol at a median of 10^6^-fold increase compared to control cells (Fig. 1b). Since *CYP24A1* expression is an established indicator of VDR activation^24^, this strengthens the conclusion that the isolated CAFs expressed a functional VDR. We further found that the mRNA expression of the cathelicidin antimicrobial peptide gene (*CAMP*), encoding the transcript for the anti-microbial peptide LL-37, was induced after VDR-engagement (median 10.3-fold) (Fig. 1b). Thus, CAFs have the capacity to respond to calcipotriol, which is in line with previous work.

It has been suggested that vitamin D_3_ analogues can restore the reactive phenotype of CAFs to a quiescent phenotype, as determined by the downregulation of *ACTA2*^15^, the gene encoding α-SMA. In line with these studies, we also found that the mRNA levels of *ACTA2* in primary CAFs cultured in conventional 2D cultures was decreased after culture with calcipotriol for 72 h (Fig. 1c). Additionally, when we analysed the effect of calcipotriol at different time points, we found that *ACTA2* expression was reduced by calcipotriol to a similar extent at 24 h and 72 h, but there was no effect at 48 h (Supplementary Fig. S1a online). The observed pattern with a decrease in the mRNA levels was not reflected at the protein level as determined by flow cytometry. In contrast, the protein expression of α-SMA was significantly increased after addition of calcipotriol for 72 h in 2D cultures (Fig. 1c). As previously shown^25^, addition of TGF-β promoted the expression of α-SMA in CAFs, and calcipotriol further increased the expression of α-SMA (Fig. 1c). The median fluorescence intensity (MFI) for α-SMA showed the same pattern (see Supplementary Fig. S1b online).

Since 2D tissue culture plastic can have an impact on the cell biology of CAFs, including cell-adhesion, paracrine signalling, activation, proliferation and gene expression, we also examined the effect of VDR stimulation in CAFs cultured in 3D collagen matrix cultures. We noted that the protein expression of α-SMA was significantly lower in CAFs cultured in 3D cultures compared to 2D cultures, supporting that plastic adherence in 2D cultures promote CAF activation. However, even in a less activated state in 3D cultures, calcipotriol did not reduce the expression of α-SMA in CAFs (Fig. 1d and Supplementary Fig. S1c online). To more closely resemble the physiological conditions of CAFs in the tumour microenvironment, we added tumour cell spheroids consisting of the pancreatic cancer cell line PANC-1 to the collagen matrix. Nevertheless, the presence of calcipotriol did not decrease α-SMA expression (Fig. 1d). The same pattern was observed when the MFI for α-SMA was analysed (see Supplementary Fig. S1c online). To further validate our flow cytometry results, we performed immunostaining analysis on the 3D collagen cultures for α-SMA on CAFs (showed in red) co-cultured with PANC-1 spheroids (stained with violet cell tracer in blue) (Fig. 1d). The α-SMA staining intensity was similar between treated and untreated cultures. Thus, in our hands, we did not find any downregulating effect of vitamin D_3_ analogues on α-SMA in CAFs in neither 2D nor 3D cultures on the protein level. On the contrary, calcipotriol promoted the protein expression of α-SMA in CAFs adherent to plastic.

The transmembrane glycoprotein podoplanin is another marker that has been shown to be expressed in the dense pancreatic stroma surrounding the tumour nests. Similar to the above observed effect on α-SMA expression, calcipotriol increased podoplanin expression in the 2D cultures (Fig. 1e), whereas TGF-β did not affect the expression of podoplanin in CAFs. In 3D cultures, the expression of podoplanin was not significantly affected by addition of calcipotriol (Fig. 1f).

**Figure 2:**
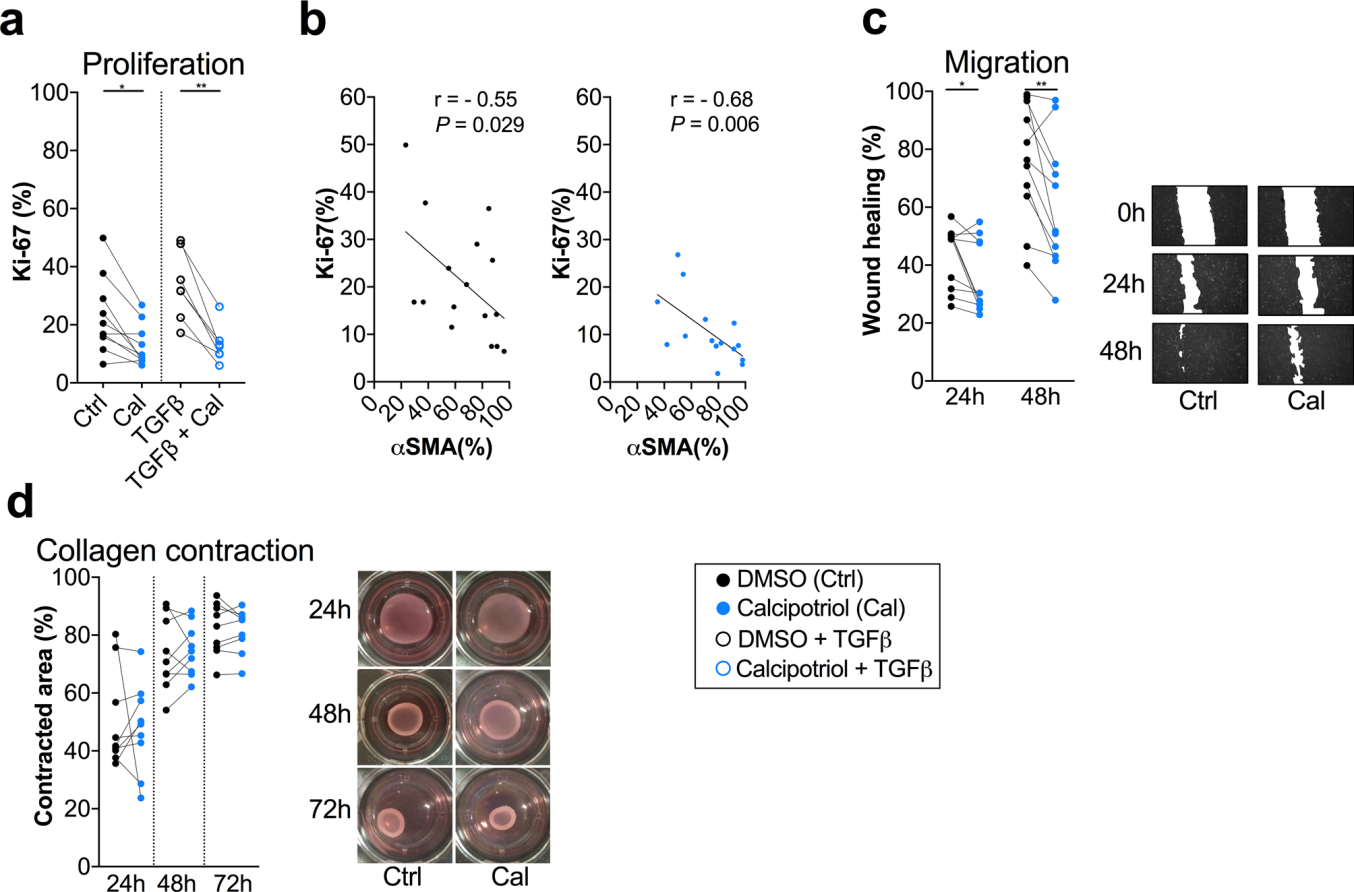
Calcipotriol diminishes proliferative and migration activity of CAFs. CAFs were treated with either DMSO control (filled circle) or 100 nM calcipotriol (blue filled circle) for 72 h. (**a**) Frequency of proliferating CAFs treated with either DMSO control, calcipotriol (*n* = 10), TGFβ (open circle) or TGFβ together with calcipotriol (blue open circle) (*n* = 7) determined by Ki-67 expression by flow cytometry. (**b**) (Left) correlation between expression of Ki-67 and αSMA in untreated CAFs and (Right) CAFs treated with calcipotriol (*n* = 16). (**c**) (Left) migration determined by wound healing assays by CAFs treated either with DMSO control or calcipotriol after 24 h and 48 h. Graph shows the percentage of the area migrated normalized to 0 h (*n* = 11). (Right) images of a representative wound healing assay (× 4 magnification) after 0 h, 24 h and 48 h. (**c**) (Left) collagen gel contraction assay after 24 h, 48 h and 72 h. The percentage of contracted area is normalized to the area at 0 h (*n* = 9). (Right) representative pictures of the gels from one donor after 24 h, 48 h and 72 h. (**a**–**c**) Lines between dots indicate paired samples. Wilcoxon matched-pairs singed rank test was used to detect statistically significant differences *p < 0.05, **p < 0.01.

We also sought to investigate other approaches by which calcipotriol could affect the activation of CAFs in 2D and 3D models. Consistent with a more quiescent phenotype, we found that calcipotriol reduced the proliferative capacity of CAFs, as determined by Ki-67 expression in 2D cultures (Fig. 2a). CAF proliferation was inversely correlated with α-SMA expression, which was even more profound in the presence of calcipotriol (Fig. 2b). In addition, scratch wound healing assays revealed that calcipotriol significantly impaired the migratory capacity of CAFs after 24 h and 48 h (Fig. 2c). Next, we performed a collagen matrix contraction assay in 3D models to evaluate the contractile capacity of CAFs and their ability to reorganize the fibre network, which could mimic the development of fibrosis in tumours. We found no significant effect of calcipotriol on the capacity of CAFs to contract the collagen matrix (Fig. 2d).

Thus, although we did not find any evidence for a decreased contractile capacity or diminished expression of α-SMA and podoplanin after VDR signalling, the proliferative and migratory ability of CAFs was decreased by calcipotriol.

### Effect of calcipotriol on CAF-derived immunoregulatory and pro-tumorigenic factors

**Figure 3:**
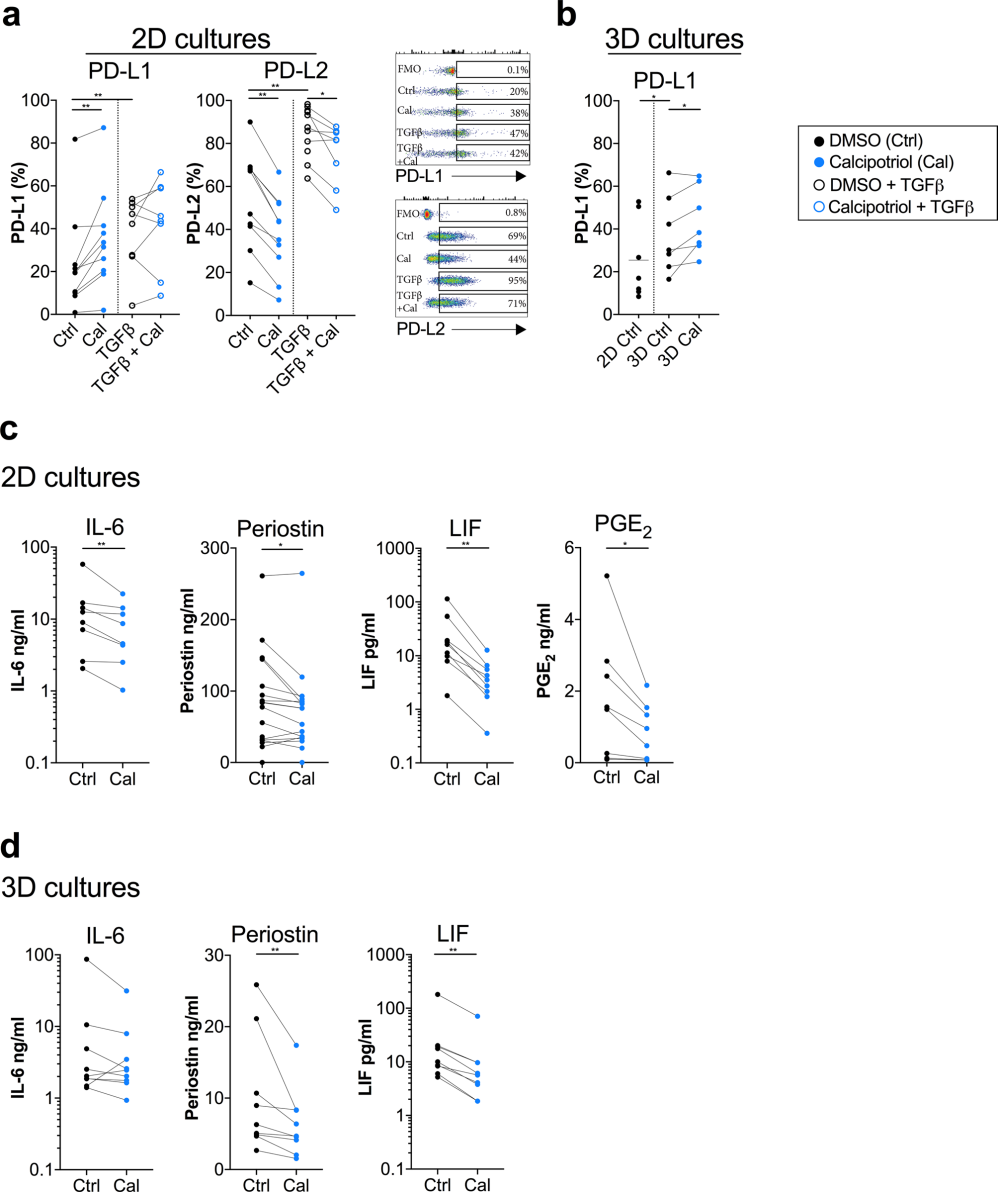
Calcipotriol reduces the release of pro-tumorigenic factors by CAFs. CAFs were treated with either DMSO control (filled circle) or 100 nM calcipotriol (blue filled circle) for 72 h. (**a**) Expression of PD-L1 and PD-L2 on CAFs cultured in 2D cultures treated with either DMSO control, calcipotriol, (*n* = 11) TGFβ (open circle) or TGFβ together with calcipotriol (blue open circle) (*n* = 8) measured by flow cytometry. Representative flow cytometry dot plots with different treatments is shown for PD-L1 and PD-L2. (**b**) Expression of PD-L1 on CAFs cultured in 2D or 3D models treated with DMSO control or calcipotriol (*n* = 7). Levels of (**c**) PGE_2_, (*n* = 8) (**c**,**d**) IL-6 (*n* = 8 and 9), periostin (*n* = 16) and LIF (*n* = 9) released by CAFs cultured in (**c**) 2D cultures and (**d**) 3D cultures and treated with either DMSO control or calcipotriol as measured with ELISA. (**a**–**d**) Lines between dots indicate paired samples. Wilcoxon matched-pairs singed rank test was used to detect statistically significant differences *p < 0.05, **p < 0.01.

Next, we investigated whether calcipotriol could modulate the expression of immunomodulatory factors known to be associated with pro-tumorigenic activities in CAFs. We have previously shown that pancreatic CAFs express the co-inhibitory ligands PD-L1 and PD-L2^23^. Notably, calcipotriol upregulated the expression of PD-L1, but on the other hand, downregulated the expression of PD-L2 on CAFs (Fig. 3a). TGF-β upregulated the expression of both PD-L1 and PD-L2 (Fig. 3a). The effect on PD-L1 expression was corroborated in 3D cultures, supporting that calcipotriol promote the expression of PD-L1 on CAFs. Furthermore, CAFs cultured in 3D collagen matrix expressed higher levels of PD-L1 compared to CAFs cultured in 2D (Fig. 3b). The expression of PD-L2 was not investigated in the 3D cultures, since initial experiments showed that collagenase, which was used to detach the CAFs from the collagen matrix, profoundly reduced the surface expression of PD-L2 (see Supplementary Fig. S2 online).

IL-6, periostin, LIF, and prostaglandin E_2_ (PGE_2_), are CAF-derived soluble factors that have been described to play a role in tumour progression^9,12,26,27^. We assessed the secretion of these factors by CAFs cultured in 2D in the presence or absence of calcipotriol using ELISA. Calcipotriol significantly downregulated the secretion of IL-6, periostin, LIF, and PGE_2_ (Fig. 3c). In 3D cultures, calcipotriol significantly reduced the production of LIF and periostin. IL-6 secretion was reduced in the majority of donors (7 out of 9) (Fig. 3d).

To summarize, calcipotriol reduced the release of several pro-tumorigenic and immunosuppressive soluble factors from CAFs. However, at the same time, it promoted the upregulation of the PD-1 ligand PD-L1, which could hamper the T cell mediated immune responses.

### Calcipotriol affect T cell effector functions in the presence of CAFs

It has previously been shown that vitamin D_3_ can directly induce growth arrest in tumour cells^15,28^. However, data also suggest that vitamin D_3_ suppress immune activation and proinflammatory responses^17^, which may act in an unfavourable manner in the context of tumour cell growth. Since CAFs have immunosuppressive capacities on T cell functions ^23^, we next examined the effects of calcipotriol on T cell activation in the absence and presence of CAFs.

**Figure 4:**
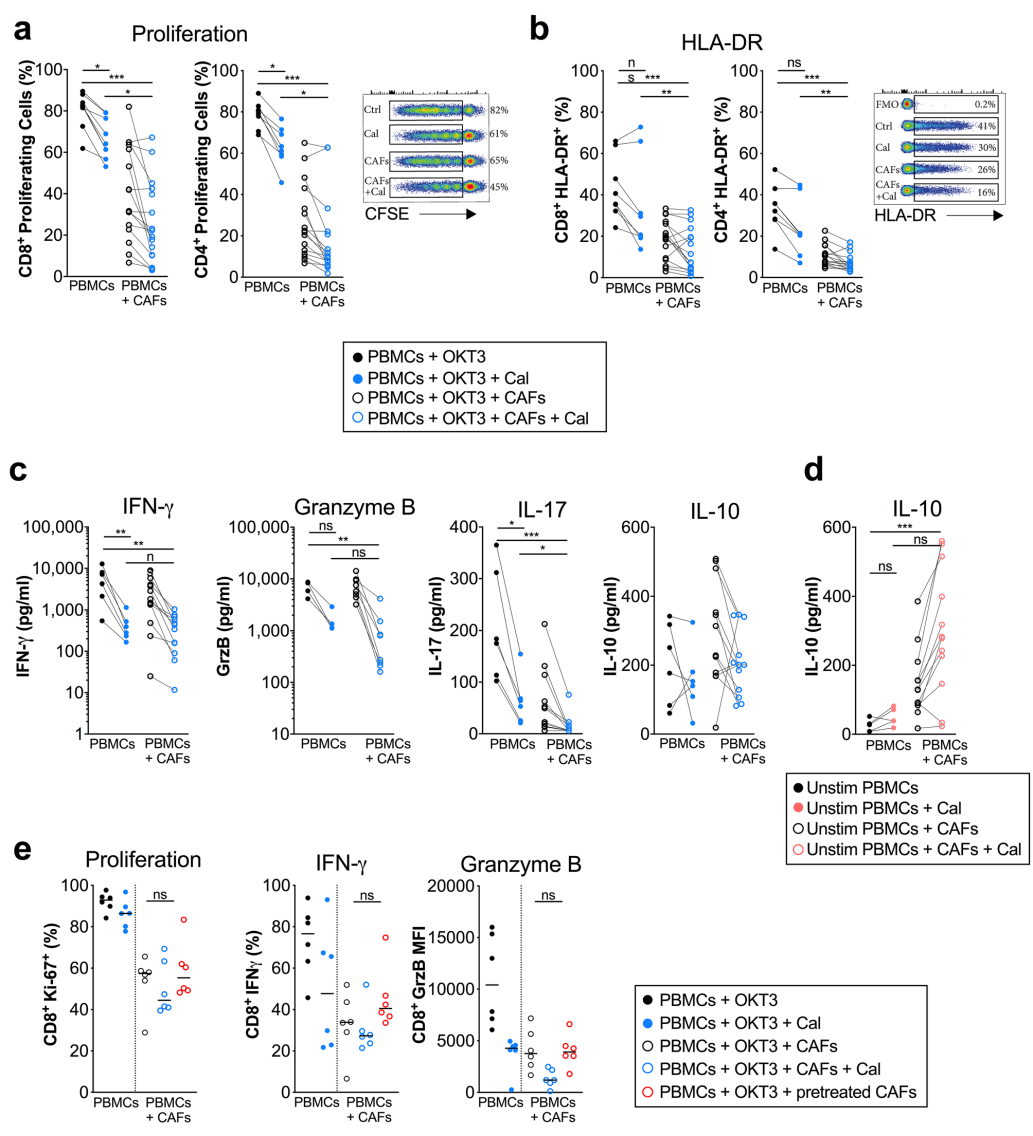
Calcipotriol exerts strong immunosuppressive effects on CD8^+^ T cells in the presence of CAFs. PBMCs were cultured alone or co-cultured with CAFs in 2D models. Cultures were treated with either DMSO control (filled circle) or 100 nM calcipotriol (blue filled circle) and PBMCs were stimulated with OKT3 (25 ng/ml) for 5 days. (**a**) Frequency of proliferating CD8^+^ and CD4^+^ T cells in the absence or presence of CAFs as determined by CFSE staining (*n* = 16). (Right) representative CFSE flow cytometry dot plots. (**b**) Expression of HLA-DR on CD8^+^ and CD4^+^ T cells in the absence or presence of CAFs measured by flow cytometry (*n* = 16). (Right) representative HLA-DR flow cytometry dot plots. (**c**) Levels of IFN-γ (*n* = 12), GrzB (*n* = 9), IL-17 (*n* = 12) and IL-10 (*n* = 12) from stimulated PBMCs cultured alone or in the presence of CAFs measured by ELISA. (**d**) Levels of IL-10 from unstimulated PBMCs cultured alone or in the presence of CAFs as measured by ELISA (*n* = 12). (**e**) For some co-cultures CAFs were pre-treated with calcipotriol for 48 h (open circle) before adding them to untreated PBMCs. PBMCs were stimulated with OKT3 (25 ng/ml) for 5 days. PMA/Ionomycin was added on day 5 for 4 h. Frequency of proliferating CD8^+^ T cells as determined by Ki-67 expression and expression of IFN-γ and granzyme B in stimulated CD8^+^ T cells as measured by flow cytometry. (**a**–**d**) Lines between dots indicate paired samples. Wilcoxon matched-pairs singed rank test or Friedman’s test were used to detect statistically significant differences for (**a**–**e**), respectively. *ns*, not significant. *p < 0.05, **p < 0.01, ***p < 0.001.

In line with other studies, we found that calcipotriol had a strong immunosuppressive effect on T cells and downregulated the proliferation of CD8^+^ and CD4^+^ T cells after stimulation with the anti-CD3 monoclonal antibody OKT3 (Fig. 4a). When both CAFs and calcipotriol were added, a further reduction in proliferation was observed. T cell inhibition by calcipotriol was also reflected by a decreased expression of the activation marker HLA-DR on CD8^+^ and CD4^+^ T cells in the presence of CAFs (Fig. 4b). Proliferation and HLA-DR expression was significantly decreased when both CAFs and calcipotriol were combined compared to when only calcipotriol was added (Fig. 4a,b). Secretion of the pro-inflammatory cytokines IFN-γ and IL-17 and the cytotoxic molecule granzyme B (GrzB) in response to stimulation was also reduced after addition of calcipotriol in the presence of CAFs (Fig. 4c). In contrast, there was no significant effect of calcipotriol on the secretion of the immunosuppressive cytokine IL-10 (Fig. 4c). However, IL-10 production was increased by calcipotriol in unstimulated conditions in the presence of CAFs (Fig. 4d). The median proportion of CD8^+^ and CD4^+^ T cells in the PBMCs used for this experiment was 43.1 (range 35.0–50.9) and 52.5 (range 46.2–63.2) percent out of CD3^+^ T cells, respectively.

In order to investigate if the calcipotriol-promoted suppression of T cells was in part mediated by altering the immunosuppressive capacity of the CAFs, we next examined how CAFs pre-treated with calcipotriol for 48 h affected subsequent T cell activation in the absence of calcipotriol. We found that pre-treated CAFs similarly affected T cell proliferation and IFN-γ and GrzB expression as compared to control CAFs without any pretreament (Fig. 4e). In summary, this suggests that calcipotriol reduces T cell activation and effector functions, which is even more profound in the presence of CAFs. However, calcipotriol does not seem to affect the immunosuppressive function of CAFs, but rather mediate the inhibitory effect directly on T cells in an additive fashion when combined with CAFs.

### Calcipotriol affect T cell effector functions in the presence of CAFs and tumour cells

**Figure 5:**
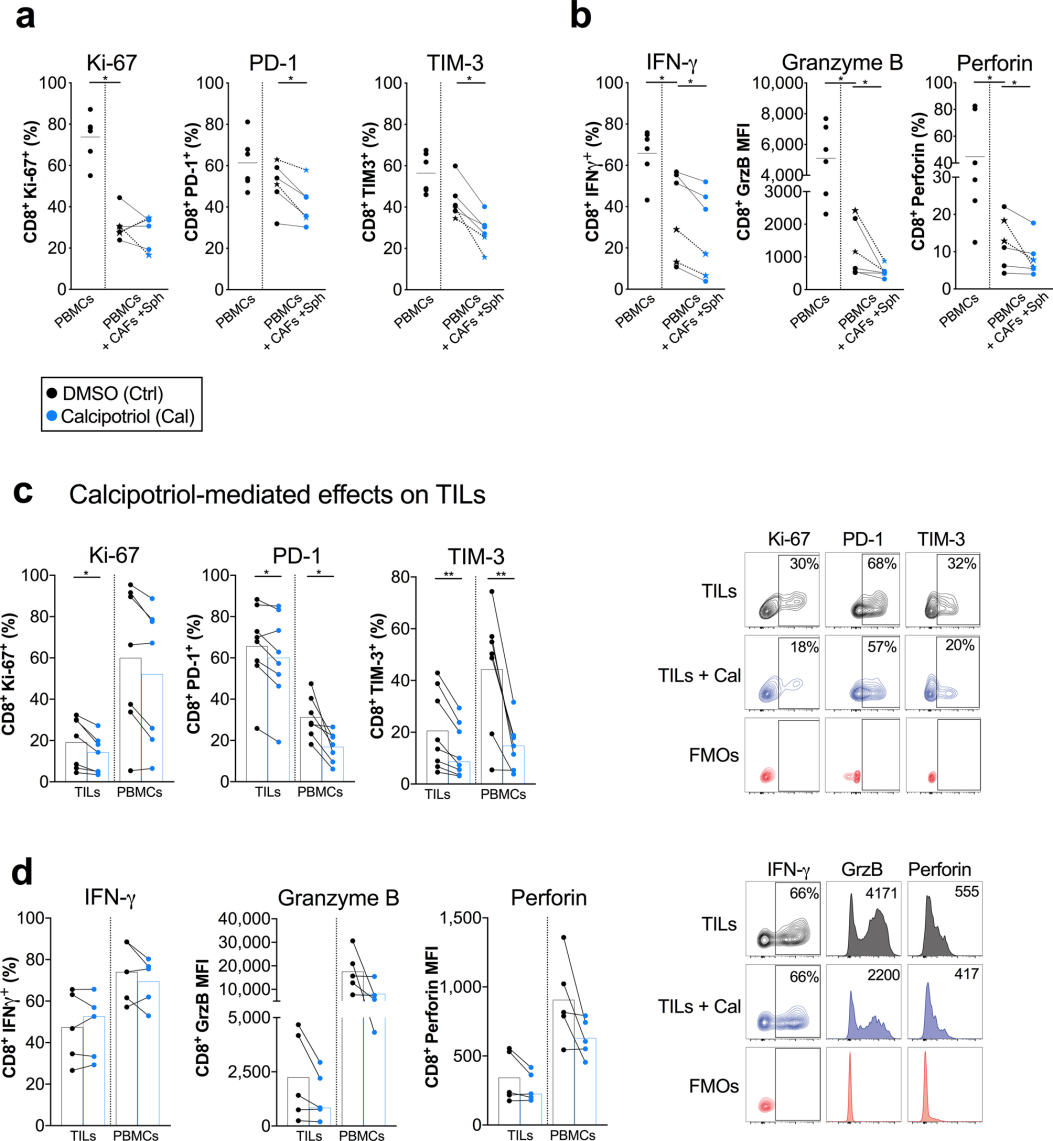
Calcipotriol exerts immunosuppressive effects on CD8^+^ T cells cultured in 3D models and on tumour infiltrating CD8^+^ T cells. (**a**,**b**) PBMCs were cultured alone or co-cultured with CAFs and PANC-1 spheroids in 3D models. Cultures were treated with either DMSO control (filled circle) or 100 nM calcipotriol (blue filled circle) and PBMCs were stimulated with OKT3 (25 ng/ml) for 5 days. PMA/Ionomycin was added on day 5 for 4 h. (**a**,**b**) Expression of (**a**) Ki-67, PD-1, TIM-3 (*n* = 6) and (**b**) IFN-γ, GrzB and perforin (*n* = 6) in stimulated CD8^+^ T cells as analysed by flow cytometry. Stars joined with dotted lines represent autologous paired samples for PBMCs and CAFs. (**c**,**d**) Cells isolated from pancreatic tumour tissues and PBMCs were treated with either DMSO control or 100 nM calcipotriol and were stimulated with OKT3 (25 ng/ml) for 3–5 days. PMA/Ionomycin was added on day 3–5 for 4 h (**c**,**d**) (Left) Expression of (**c**) Ki-67 (*n* = 7), PD-1, TIM-3 (*n* = 8) and (**d**) IFN-γ, GrzB and perforin (*n* = 5) in stimulated CD8^+^ tumor-infiltrating T cells as measured by flow cytometry. (Right) representative flow cytometry dot plots and histograms showing stimulated TILs in black and stimulated TILs treated with calcipotriol in blue. FMOs are shown in red. (**a**–**d**) Lines between dots indicate paired samples, histograms indicate the median. Wilcoxon matched-pairs singed rank test was used to detect statistically significant differences *p < 0.05.

We next investigated how T cells are affected by calcipotriol in 3D cultures consisting of both CAFs and PANC-1 spheroids. Even though the proliferation was not significantly suppressed by calcipotriol in this setting, the co-inhibitory markers PD-1 and TIM-3 were downregulated in CD8^+^ T cells (Fig. 5a). Moreover, the proportion of CD8^+^ T cells expressing IFN-γ, GrzB and perforin upon phorbol 12-myristate 13-acetate and ionomycin (PMA/I) stimulation was significantly reduced after addition of calcipotriol (Fig. 5b). The same pattern was seen when the experiments were performed with autologous T cells and CAFs (shown with stars) (Fig. 5a,b).

### Effects of calcipotriol on T cells isolated from pancreatic cancer tissues

Next, we evaluated the effects of calcipotriol on tumour infiltrating lymphocytes (TILs) isolated from pancreatic tumour tissues as well as on PBMCs from the same patients. The proportion of CD3^+^ T cells within the lymphocyte gate and the percentage of CD8^+^ and CD4^+^ out of CD3^+^ T cells of the isolated TILs and PBMCs are shown in Supplementary Fig. S3 online. The expression of PD-1 on CD4^+^ and CD8^+^ T cells was higher in the TILs compared to PBMCs (see Supplementary Fig. S3 online). Cells were cultured in the presence or absence of calcipotriol and stimulated with OKT3 for 3–5 days. Some cultures (n = 5) were restimulated with PMA/I for the last 4 h to study expression of IFN-γ and cytotoxic molecules. In line with the 3D co-cultures, calcipotriol inhibited TIL proliferation and downregulated the expression of the co-inhibitory markers PD-1 and TIM-3 (Fig. 5c). A reduction in GrzB and perforin expression was observed in both CD8^+^ TILs and patient PBMCs upon calcipotriol treatment in 4 out of 5 donors for GrzB and in 3 out of 5 donors for perforin in TILs. However, the proportion of CD8^+^ TILs cells expressing IFN-γ was not changed by calcipotriol (Fig. 5d). In summary, calcipotriol seemed to diminish T cells activation and functionality in both 3D models and patient-derived TILs.

## Discussion

In pancreatic cancer, the stroma can account for about 80% of the total tumour mass^3^ and is considered the main limitation for the highly unsatisfactory treatment outcomes^3–6,12^. The major cellular type in the stroma are CAFs, which are key contributors to cancer progression, due to their capacity to interact with cancer cells and other stromal components such as immune cells. However, the functional heterogeneity of CAFs within the tumour presents a challenge to target them. Emerging therapeutic approaches with vitamin D_3_ analogues aim at reprogramming the activated state of CAFs to a quiescent phenotype, leading to stroma remodelling and improved survival^15^. It has also been shown that CAFs present a phenotypic plasticity, allowing them to switch between pro- and anti-tumorigenic phenotypes^8^. Here, we show that pancreatic human primary CAFs respond to the vitamin D_3_ analogue, calcipotriol, promoting differentiation towards a less tumour supportive CAF phenotype. However, calcipotriol also has an immunosuppressive effect on T cells, which is even stronger in the presence of CAFs. Thus, although VDR stimulation may have an advantageous effect on the phenotype of pancreatic CAFs, a potential T cell mediated response against tumour cells may at the same time be dampened by vitamin D_3_.

The stroma and the CAFs surrounding the pancreatic tumour cells display heterogeneity and may have both protective and tumour-promoting functions. Several phenotypes have recently been described in the literature. α-SMA^high^ myofibroblasts are generally localized in close proximity with tumour cells^8^, which have been suggested to restrain tumour cell growth and migration^7^. Inflammatory α-SMA^low^ cells are located more distantly from the tumour nests, and are associated with elevated expression of tumour promoting cytokines, including IL-6^8^. Tumour-derived TGF-β has been described to promote differentiation into myofibroblasts, whereas IL-1 signalling via JAK/STAT activation is driving the differentiation into inflammatory CAFs^29^. Another recent study identified four different subpopulations of pancreatic CAFs with different expression patterns of podoplanin, periostin, and myosin-11, which were associated with prognosis^11^. High expression of the gene encoding periostin (POSTN) was associated with poor survival, whereas membrane expression of podoplanin was related to better prognosis.

We found that VDR signalling increased the expression of α-SMA and podoplanin in CAFs, whereas the secretion of IL-6, LIF, and periostin was reduced. To put our findings in the context of the above studies on CAF heterogeneity and prognosis, our data suggest that vitamin D_3_ overall promote less tumour supportive CAFs.

Consistent with a more anti-inflammatory phenotype, we also found that calcipotriol decreased the capacity of CAFs to proliferate and migrate. This is in accordance with the observed reduction of the pro-tumorigenic factors IL-6, periostin, LIF and, PGE_2_ which previously have been shown to promote tumour cell growth^8–12,26,27^. Notably, we also showed that calcipotriol did not affect the contractile ability of CAFs, as measured by collagen contraction assays, which mimics the development of fibrosis in tumours. This result is in contrast to the study by Sherman et al*.*^15^ who showed that VDR signalling reduces fibrosis in a mice pancreatitis model. Treatment with calcipotriol could potentially reverse the phenotype of CAFs, resulting in a diminished paracrine signalling, in turn hampering tumour cell migration and invasion. However, further investigation is required to confirm the direct consequences of the downregulation of these factors on tumour cells.

Previous studies have shown that pancreatic stellate cells can adequately respond to vitamin D_3_ analogues by the downregulation of the gene encoding α-SMA, *ACTA2*. Here, we also show a downregulation in expression of the *ACTA2* gene in response to calcipotriol at 24 h and 72 h. Others have previously shown a downregulation in *ACTA2* expression in human pancreatic CAFs in response to VDR ligation after 48 h^15^, but for unknown reasons, we found no significant effect on *ACTA2* expression at this time point. Nevertheless, we found no correlation between mRNA levels and protein abundance but showed that the actual protein levels for α-SMA were in fact upregulated by calcipotriol at 72 h in 2D cultures. In line with previous studies using the same α-SMA antibody clone, we found an increased expression of α-SMA in response to the profibrogenic cytokine, TGF-β, suggesting that the α-SMA staining is reliable^25,30^. A possible explanation for this apparent paradox is that the protein levels depend on different transcriptional and post-transcriptional processes as well as translational rates and protein stability. Thus, changes in gene transcription can often not be correlated to the protein level^31–33^.

The expression of α-SMA in cancer is often used to identify pathologic myofibroblasts whose expression has been positively correlated with an increased contractile and fibrogenic activity^34^, as well as with a dismal clinical prognosis^11,35^. However, deletion of α-SMA^+^ CAFs in pancreatic cancer has been shown to induce cancer progression instead of its suppression^7^. Recent studies with experimental models of organ fibrosis^36,37^ and oral carcinoma^38^ also show no correlation with α-SMA expression and the contractile ability of fibroblasts. In line with the study by Öhlund et al*.* suggesting that α-SMA^high^ CAFs display an anti-inflammatory phenotype, other studies have found that over-expression of α-SMA attenuates the proliferative activity of CAFs^30,38^. We also show a negative correlation between Ki-67 and α-SMA expression both in the presence and absence of calcipotriol. These controversial outcomes together with large complexity of different CAF subpopulations, suggests that the actual role of α-SMA^+^ CAFs in cancer need to be reconsidered.

In a previous study, we showed that CAFs induce the expression of co-inhibitory markers on T cells, and inhibit T cell proliferation and functionality, and that blockade of PGE_2_ and PD-L1/L2 partially reverted T cell proliferation^23^. Here we studied the impact of calcipotriol on expression of immunomodulatory molecules on CAFs and their immunosuppressive action on T cells. We found that expression of PD-L1 was upregulated in CAFs in response to vitamin D_3_, suggesting that elevated vitamin D_3_ signalling can suppress anti-tumour immunity via PD-1. This is consistent with the findings by Dimitrov et al*.* who showed that human, but not murine, epithelial and myeloid cells increase the expression of PD-L1 upon VDR signaling^39^. They further found that vitamin D-mediated suppression of T cells could be reverted by PD-L1 neutralization. Vitamin D also upregulated the gene encoding PD-L2 (*CD273*) in myeloid, but not epithelial cells, although the protein expression was not examined in this study. In contrast, we found that surface expression of PD-L2 in CAFs was decreased in response to VDR signalling. PD-L1 and PD-L2 compete for binding to PD-1, and PD-L2 has a higher affinity to PD-1 compared to PD-L1^40^. Thus, the increased expression of PD-L1 may be counteracted by the decrease in PD-L2 expression to compensate for the relative effect on PD-1-mediated T cell blockade. PD-L2 expression on CAFs has also previously been found to be the dominant PD-1 ligand in murine models of lung cancer, leading to killing of antigen-specific T cells^41^.

However, since PD-L1 blockade can revert vitamin D-mediated inhibition of T cells^40,42^ it is possible that the increased expression of PD-L1 on CAFs by calcipotriol is involved in suppressing CD8^+^ T cell activity via PD-1 engagement.

In our 2D cultures and in line with previous studies, the vitamin D_3_ analogue strongly suppressed CD8^+^ T cell proliferation and functionality and increased the production of the immunosuppressive cytokine IL-10 from unstimulated PBMCs. It has been shown that vitamin D promote tolerogenic dendritic cells, which in turn induce CD4^+^CD25^+^Foxp3^+^ and CD4^+^IL-10^+^ regulatory T cells in a mice model^43^. COX2 inhibition restored the CAF-mediated suppression of T cells^23^, suggesting that PGE_2_ is important for inhibiting T cell activation in this setting. Although we show that calcipotriol decreased the secretion of PGE_2_ from CAFs, VDR signalling failed to rescue T cells from CAF-mediated suppression. In fact, the combination of both CAFs and calcipotriol appeared to further potentiate the inhibitory effect on T cell proliferation and partly also on T cell function. These observations arise the question whether the vitamin D_3_ analogue can affect the immunoregulatory function of CAFs. In order to examine if calcipotriol is acting on both the CAFs and the T cells to mediate the immunosuppressive effect on T cells, we examined how CAFs that were pre-treated with calcipotriol before addition to the cultures would affect subsequent T cell activation. Calcipotriol-treated CAFs suppressed T cells to a similar degree as compared to untreated control CAFs, suggesting that calcipotriol mediates its effect directly on T cells and not by influencing the immunosuppressive activity of CAFs. However, it can also be speculated that the potential calcipotriol-mediated effect on CAFs is lost during the five-day culture in the absence of calcipotriol. Together, our data suggests that calcipotriol do not have a synergistic effect on the inhibitory activity of CAFs, but that there is an additive effect on the suppression of T cells when combined.

In recent studies, the scientific community has acknowledged the advantages of using 3D cell culture models over 2D cultures. The 3D models have a more accurate representation of the tissue environment, a more appropriate way to study cellular responses and interactions, as well as a better approach to mimic the in vivo cell fate and functionality. Here, we used 3D collagen cell culture models to better mimic cell interactions between CAFs, tumour cells and T cells. We found that α-SMA was downregulated in 3D cultures compared to 2D cultures, which could indicate a reversion of the activated phenotype, triggered by the plastic surface of the cell culture plates, to a more quiescent phenotype. Podoplanin expression was similar in 2D and 3D cultures, which could suggest that the expression of this marker is less affected by plastic adherence. Even though cells adopted a more quiescent phenotype in 3D cultures, they still responded to calcipotriol by downregulating the release of the pro-tumorigenic factors IL-6, periostin, and LIF in line with our results from the 2D cultures. However, in contrast to the 2D cultures, calcipotriol did not have any significant effect on α-SMA and podoplanin expression in CAFs. We further showed that CAFs and PANC-1 spheroids dampened the proliferative activity and functionality of CD8^+^ T cells, and when calcipotriol was added to the co-cultures, the suppressive effects were accentuated to an even greater degree. To confirm whether these results resemble the native tumour microenvironment, we isolated cells from human pancreatic tumour tissues and treated it with calcipotriol. We showed that calcipotriol had similar effects on CD8^+^ tumour infiltrating T cells as in the 3D and 2D models, hampering their activation by downregulating proliferation, PD-1 and TIM-3 expression, and by partially inhibiting their functionality. Thus, although differences in CAF activation markers in response to calcipotriol could be detected between 2 and 3D cultures, a reduction in secretion of pro-tumorgenic factors from CAFs as well as T cell functions were observed in both experimental setups.

Another aspect to consider when studying the VDR-signalling is the lack of conservation in vitamin D_3_ response elements in non-primates. Many of the genes that are activated in human cells after VDR-signalling are not induced in murine cells^42,44^. For instance, the anti-microbial peptide human beta-defensin 2 and the pattern recognition receptor NOD2 are induced in human cells, but not in murine cells upon vitamin D stimulation, which leads to a boost of the innate immune response during a pathogen threat in humans but not in mice^42^. In the same way, vitamin D_3_ can induce production of the anti-microbial peptide cathelicidin, which when cleaved to the active form LL-37 is involved in host defence and immunomodulation. High levels of LL-37 has been associated with cancer stem cell growth and survival and pro-angiogenic effects in PDAC^44^. Since the gene encoding LL-37 is controlled by VDR activation in humans, but not in mice, the positive effects of vitamin D_3_ observed in murine models of PDAC should be interpreted with caution. Due to the importance of the adaptive immune system in cancer surveillance, it can be speculated that 2D or 3D cell culture models could be a better approach to study the effects of vitamin D_3_ on the tumour microenvironment.

The favourable outcomes of vitamin D_3_ analogues in cancer have mainly focused on cancer cells due to its role in cell cycle arrest, inhibition of the cells invasive capacity^45,46^, and of the anti-angiogenic activity^47,48^. However, even though this seems to be beneficial in the early stages of cancer, it has been shown that during tumour progression cancer cells can downregulate VDR and become resistant to vitamin D complements^49^. A recent study of pancreatic cancer reported unfavourable outcomes of vitamin D analogues on cancer cells derived from PDAC quasi-mesenchymal subtype, inducing migration and development of metastasis^50^. Therefore, depending on the stage and cancer subtype, vitamin D supplements may not only be ineffective but could possibly have negative effects, although this needs to be confirmed in larger clinical trials before any firm conclusions can be drawn.

T cells are key factors in the adaptive immune system and CD8^+^ T cells are the main effector cells in tumour surveillance. Therefore, during the last decade, immunotherapy drugs have offered significant benefit in cancer treatment due to their novel role in reinforcing the patients’ effector T cells to kill cancer cells. Our results suggest that VDR signalling dampens the adaptive immune system, which may jeopardize the anti-tumour immunity of the patient and, hence, could potentially counteract the effect of immunotherapies. However, ongoing and future clinical trials using both PD-1 blockade and vitamin D analogue treatment will determine if vitamin D can shift CAFs to a more anti-inflammatory and anti-tumorigenic phenotype and if checkpoint blockade can promote T cell mediated anti-tumour responses in pancreatic cancer.

## Material and methods

### Patient samples

Pancreatic tumour tissues were collected from 19 patients undergoing surgery at the Upper Gastrointestinal Diseases Unit of Karolinska University Hospital, Huddinge, Sweden as previously described^23^. Peripheral blood samples were collected from patients and healthy blood donors. All research was performed in accordance with relevant guidelines and regulations. Written informed consent was obtained from all participants. All patients were over 18 years old. The study and all experimental protocols were approved by the regional ethical review board in Stockholm, Sweden (entry nos. 2013/977-31.3 and 2017/722-32, 2018/1792-31/2).

### Cell isolation

Cancer-associated fibroblasts (CAFs) were isolated from freshly resected pancreatic tumour tissues as previously described^23^. Briefly, tissues were cut into small pieces and cultured in 6 well plates in Dulbecco’s modified Eagle’s medium (DMEM) (GE Healthcare, South Logan, UT, USA) supplemented with 10% foetal bovine serum (PAA laboratories GmbH, Pasching, Austria) and 100U/ml of penicillin and 100 μg/ml streptomycin (PEST) (HyClone, South Logan, UT, USA) (complete DMEM) under standard culture conditions (37 °C, 5% CO_2_). CAFs were let to migrate out the tissue fragments, expanded up to passage 3–4 and cryopreserved until analysis.

The phenotype of the isolated CAFs has previously been published by our group^23^. In summary, CAFs were positive for the stromal markers CD29, CD44, CD73, CD90, CD105 and partially positive for PD-L1, PD-L2 and αSMA. CAFs were also negative for the endothelial marker CD31 and the epithelial marker EPCAM. For isolation of tumour infiltrating lymphocytes, tumour tissue pieces (mean of 160 mg) were cut into small pieces with a scalpel and cells were released by mechanical disaggregation using a GentleMACS dissociator (Miltenyi Biotec, Bergisch Gladbach, Germany). The cell suspension was then filtered through 70 μm cell strainer (VWR, Radnor, PA), washed and cryopreserved until analysis. Peripheral blood mononuclear cells (PBMCs) were isolated from patients and healthy donors by density gradient over Lymphoprep gradient (#1114547, Axis Shield, Dundee, UK).

### Vitamin D_3_ analogue

The vitamin D_3_ analogue, calcipotriol, (Tocris, Abingdon, UK) was dissolved in DMSO and stock solutions of 100 μM were stored at − 20 °C.

### CAFs cultures in 2D

CAFs were cultured in 24-well plates at a concentration of 25 × 10^5^ cells/well under standard culture conditions. Cells were let to attach overnight and treated with calcipotriol (100 nM) or DMSO for 72 h and analysed by flow cytometry. For a set of experiments 10 ng/ml TGF-β (#MAB246, R&D Systems, Minneapolis, MN, USA) was also added.

### Real-time qPCR

To determine gene expression on CAFs, cells were cultured in 6-well plates with or without calcipotriol for 24, 48 and 72 h. RNA was isolated with DNA/RNA Mini Kit (Qiagen, Hilden, Germany) according to the manufacturer’s instructions and used to synthesize cDNA using SuperScript VILO cDNA synthesis kit (Thermo Fisher Scientific, Waltham, MA, USA), which was then used for qPCR. Transcripts of *VDR*, *CYP24A*, *CAM*P, *ACTA2*, and the house keeping gene *18S* rRNA were amplified using SYBR green (Thermo Fisher Scientific) mixed with forward/reverse primers (see Supplementary Table S1 online) and measured using real-time qPCR (CFX96 Real-Time PCR Detection Systems, Bio-Rad) at the following conditions:

*VDR*, *CYP24A,* and *CAMP*: 92 °C for 10 min, 45 cycles at 95 °C for 15 s and 65 °C for 1 min. *18S* and *ACTA2*: 95 °C for 10 min, 45 cycles at 95 °C for 15 s and 60 °C for 1 min. Data were analysed using the delta-delta Ct method and presented as fold change.

### Spheroids

PANC-1 spheroids were established as previously described by others^51^. Cells were first cultured under normal conditions in T75 flasks in complete DMEM medium. Then, the cells were trypsinated, counted and cultured in non-tissue culture treated 96-well plates at a concentration of 2500 cells/well in 100 μL complete DMEM medium supplemented with methylcellulose (#M0512, Sigma-Aldrich, Saint Louis MO, USA) at a final concentration of 0.24%. Cells were let to form spheroids for 4 days under standard culture conditions before using them in 3D cultures. The stock solution of methylcellulose was prepared following previously published protocols^51^.

### 3D models and cultures

Collagen matrices were prepared by adapting a protocol published by others^52^. Primary fibroblasts at a concentration of 1 × 10^5^ cells/matrix were mixed with PureCol bovine type I collagen (#5005, Advanced Biomatrix, San Diego, CA, USA), and 5 × Dulbecco's modified Eagle's medium (DMEM) (#31600-083, Thermo Fisher Scientific) adjusted to a pH of 7.2 and supplemented with L-glutamine (#SH30034.01, Hyclone), NaHCO_3_, FBS and PEST with a final concentration of 2 mg/ml of collagen I. The amount of the reagents added can be found as Supplementary Table S2 online.

Two hundred μL of the acellular collagen layer was added to non-tissue culture treated 24-well plates. After 30 min, 600 μL of the cellular collagen layer was added on top of the acellular layer and let to polymerize for 2 h. After polymerization, the matrices were separated from the walls with a pipette tip and complete DMEM medium with 100 nM calcipotriol or DMSO was added. The matrices were incubated for 72 h under standard culture conditions and CAFs were isolated and analysed by flow cytometry.

In a set of experiments, 20 PANC-1 spheroids per well were added to the cellular collagen layer. To distinguish CAFs from PANC-1 by flow cytometry, CAFs were stained with cell tracer violet (#C34557, Molecular Probes, Life Technologies, CA, USA) (3 μg/ml) in PBS at 37 °C for 10 min prior to adding them to the matrix.

To isolate CAFs, matrices were cut into small pieces and digested with 0.25 mg/ml of Collagenase IV (#C5138, Sigma-Aldrich) and 0.25 μg/ml DNase I (#10104159001, Sigma-Aldrich) in PBS, in a 37 °C water bath under gentle shaking. After 5–10 min, cold 5 mM EDTA in PBS was added to the digestion solution and cells were filtered through 70 μm cell strainer.

### Contraction assays

3D models with CAFs were made in duplicates and pictures of the matrix were taken after 24, 48 and 72 h. The area of the matrix was measured by Image J (NIH, Bethesda, MA, USA). Data are expressed as percentage of contracted area normalized with the area of the gel at 0 h.

### Immunofluorescence

The cells in collagen matrices were fixed for 20 min with 4% paraformaldehyde and 0.5% glutaraldehyde in PBS and washed twice with PBS. Prior to blocking (1% BSA and 4% goat serum in 0.1% Tween-20-PBS), the cells were permeabilized with 0.3% X-100 Triton in PBS for 10 min and washed three times with PBS. Unconjugated anti-αSMA (mouse monoclonal, #14-9760-82, Thermo Fisher Scientific, dilution 1:100) antibody was used as primary antibody. Goat anti-mouse secondary antibody (dilution 1:1000) was used for fluorescence detection. For detection of PANC-1 spheroids, cells were stained with violet cell tracer prior to adding them in the collagen matrix. An Olympus IX71 fluorescent microscope was used, and pictures were taken with Olympus DP71 camera. The data was collected and edited using Cellsens Dimension software (Olympus, Tokyo, Japan).

### Scratch wound healing assays

CAFs were plated in 6-well plates and grown up to 90% confluence. Then 10 μL pipette tip was used to make a scratch. Cultures were treated with calcipotriol (100 nM), or DMSO. Pictures were taken at 24 h and 48 h and the wounded area was analysed by Image J. Data are expressed as percentage of the wounded area normalized to the initial wounded area.

### Proliferation assays in 2D and 3D cultures

For 2D cultures, PBMCs from healthy donors were labelled with carboxyfluorescein succinimidyl ester (CFSE) (#C34554, Molecular Probes, Life Technologies) (5 μg/ml) in PBS at 37 °C for 15 min and washed cells were plated in 24-well plates (1 × 10^6^) in the presence or absence of 30 Gy irradiated CAFs at a 1:10 ratio (1 CAF per 10 PBMCs). The culture medium used was RPMI-1640 (GE Healthcare, South Logan, UT, USA) supplemented with 10% human AB serum and PEST.

The cell suspension isolated from pancreatic cancer tissues was plated in 96-well round bottom plates with calcipotriol or DMSO. For 3D cultures, PBMCs from healthy donors (1 × 10^6^) were added into the collagen layer with or without CAFs and PANC-1 spheroids. The culture medium used was RPMI-1640 supplemented with 10% FBS and PEST. PBMCs in 2D and 3D models were stimulated with OKT3 (25 ng/ml) (#317315, Biolegend) and treated with calcipotriol (100 nM) or DMSO at the start of the culture. For a set of experiments, CAFs were pre-treated with calcipotriol for 48 h before adding them to the PBMCs. On day 5, PBMCs were directly harvested or isolated with collagenase from the gel and analysed by flow cytometry. For intracellular detection of cytokines, PBMCs were stimulated and treated with 25 ng/ml PMA, 1 μg/ml ionomycin, 10 μg/ml Brefeldin-A (#79346, #I0634 and #B7651 Sigma-Aldrich,), and Golgi Stop (diluted 1:625, #554715, BD) on day 4–5 for 4 h.

### Flow cytometry

Harvested or isolated CAFs and PBMCs from 2 and 3D cultures were washed in FACS buffer (2 mM EDTA, 0.2% bovine serum albumin) and stained in 96-well plates with appropriate monoclonal antibodies (see Supplementary Table S3 online). For extracellular staining, 7AAD was used to distinguish live from dead cells. For intracellular staining, cells were fixed with BD Cytofix/Cytoperm kit according to manufacturer’s instructions. Fixability viability stain was used to discriminate live from dead cells according to manufacturer´s instructions. FACSCanto II (BD) was used for data acquisition and FlowJo (BD) version 10.6.1 was used for data analysis.

### ELISAs

The levels of PGE_2_ (#514010, Cayman Chemical, Ann Arbor, MI, USA), periostin (#DY3548B, R&D systems), LIF (#DY7734-05, R&D systems), IFN-γ (#3420-1H-6, Mabtech), and Granzyme B (#3585-1H-6, Mabtech) in cell culture supernatants were measured with ELISA Kits according to manufacturer´s instructions. For IL-6, IL-10 and IL-17 an in-house ELISA was set up, using a monoclonal anti-human capture antibody #MAB206, #MAB127107 and #MAB317 (R&D Systems), a standard of the recombinant human antibody #206-IL, #1064-IL, #317IL (R&D systems), and a biotinylated anti-human antibody #BAF206, #BAF217, #BAF317 (R&D systems), accordingly. A streptavidin conjugated to poly-horseradish peroxidase was used for all the enzymatic reaction (#M2032, Sanquin, Amsterdam, Netherlands).

### Statistical analysis

The data was analysed by Wilcoxon matched-pairs signed rank test when two paired groups were compared or with Friedman’s test followed by Dunn’s multiple comparison test for differences between three groups. Spearman’s correlation test was used to analyse the relationship between different variables. All statistical analysis was done using GraphPad Prism version 8.00 (La Jolla CA, USA). A *p* value < 0.05 was considered statistically significant.

## Supplementary information

Supplementary information.

## Data Availability

The datasets generated during and/or analysed during the current study are available from the corresponding author on reasonable request.
