# Supplementary material for: The vitamin D analogue calcipotriol promotes an anti-tumorigenic phenotype of human pancreatic CAFs but reduces T cell mediated immunity
Source: Sci Rep. 2020 Oct 15;10:17444. doi: 10.1038/s41598-020-74368-3 (PMC7562723; doi:10.1038/s41598-020-74368-3)
Supplement: Supplementary file 1 — Supplementary information. [file 41598_2020_74368_MOESM1_ESM.pdf]

Supplementary material and methods

The vitamin D analogue calcipotriol promotes an anti-tumorigenic phenotype of human pancreatic CAFs but reduces T cell mediated immunity

Laia Gorchs[1*], Sultan Ahmed[1], Chanté Mayer[1], Alisa Knauf[1], Carlos Fernández Moro[1,2], Mattias Svensson[3], Rainer Heuchel[4], Elena Rangelova[4,5], Peter Bergman[1,6], Helen Kaipe[1,7*]

[1]*Department of Laboratory Medicine, Karolinska Institutet, Stockholm, Sweden,*
[2]*Department of Pathology/Cytology, Karolinska University Hospital, Stockholm, Sweden*
[3]*Department of Medicine, Centre for Infectious Medicine, Karolinska University Hospital, Stockholm, Sweden*
[4]*Department of CLINTEC, Karolinska Institutet, Stockholm, Sweden,*
[5]*Pancreatic Surgery Unit, Centre for Digestive Diseases, Karolinska University Hospital, Stockholm, Sweden*
[6]*Infectious Disease Clinic, The Immunodeficiency Unit, Karolinska University Hospital, Stockholm, Sweden*
[7]*Clinical Immunology and Transfusion Medicine, Karolinska University Hospital, Stockholm, Sweden*

Supplementary Figure S1

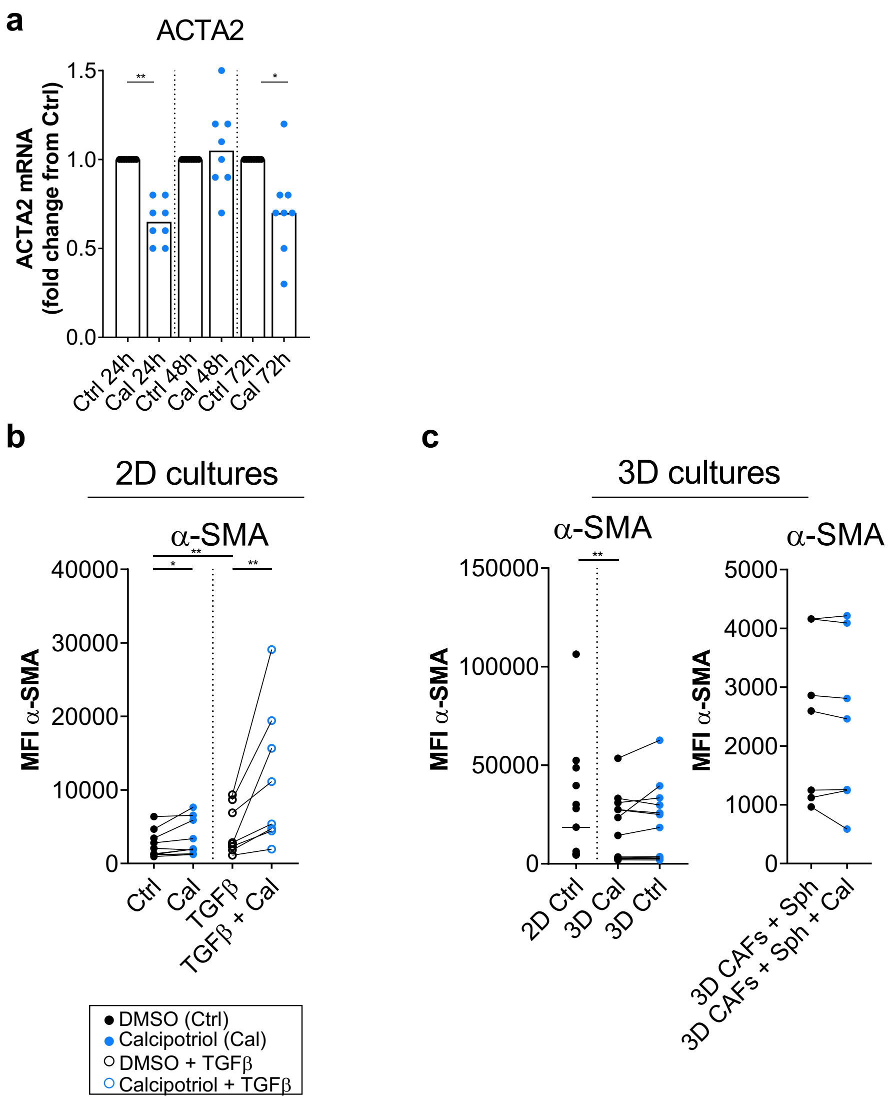

Supplementary Figure S1. Effect of calcipotriol treatment on ACTA2 gene and α-SMA protein expression in CAFs. CAFs were treated with either DMSO control (●) or 100 nM calcipotriol (●). **(a)** Gene expression of *ACTA2* was analysed in CAFs by qPCR after treatment with calcipotriol (Cal) for 24, 48 and 72 h and the data is presented as fold change normalized to the DMSO control (Ctrl). **(b)** Mean flouresence intensity (MFI) of α-SMA in CAFs treated with either DMSO control, calcipotriol, (*n* = 11) TGFβ (o) or TGFβ together with calcipotriol (o) at 72 h (*n* = 8) in 2D cultures. **(c)** (left) MFI of αSMA in CAFs cultured in 2D or 3D models treated with either DMSO control or calcipotriol (*n* = 13). (right) Expression of αSMA on CAFs co-cultured with spheroids in 3D models (*n* = 7). Wilcoxon matched-pairs singed rank test was used to detect statistically significant differences *P<0.05, **P<0.01, ***P<0.001.

Supplementary Figure S2

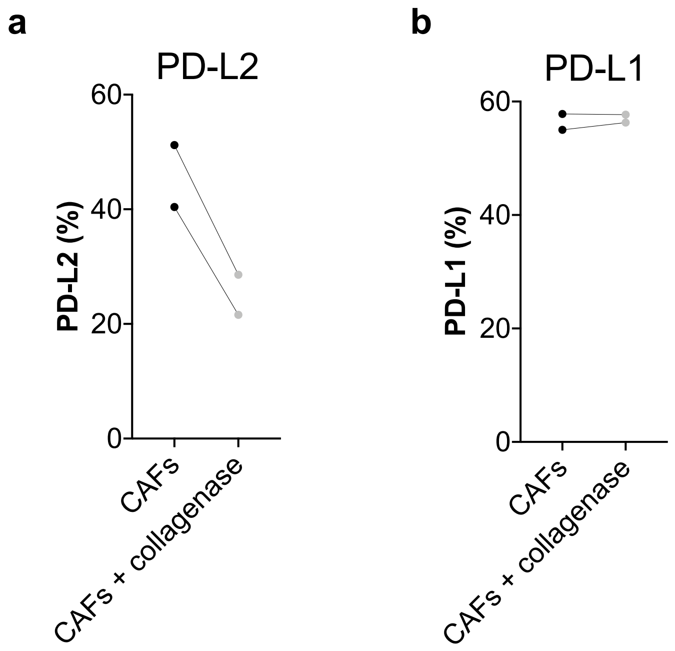

Supplementary Figure S2. Collagenase decreases the expression of PD-L2. Cultured CAFs on plastic wells were trypsinated and treated or not with collagenase for 5 minutes. (**a**) PD-L2 and (**b**) PD-L1 expression was determined by flow cytometry. Lines between dots indicate paired samples.

Supplementary Figure S3

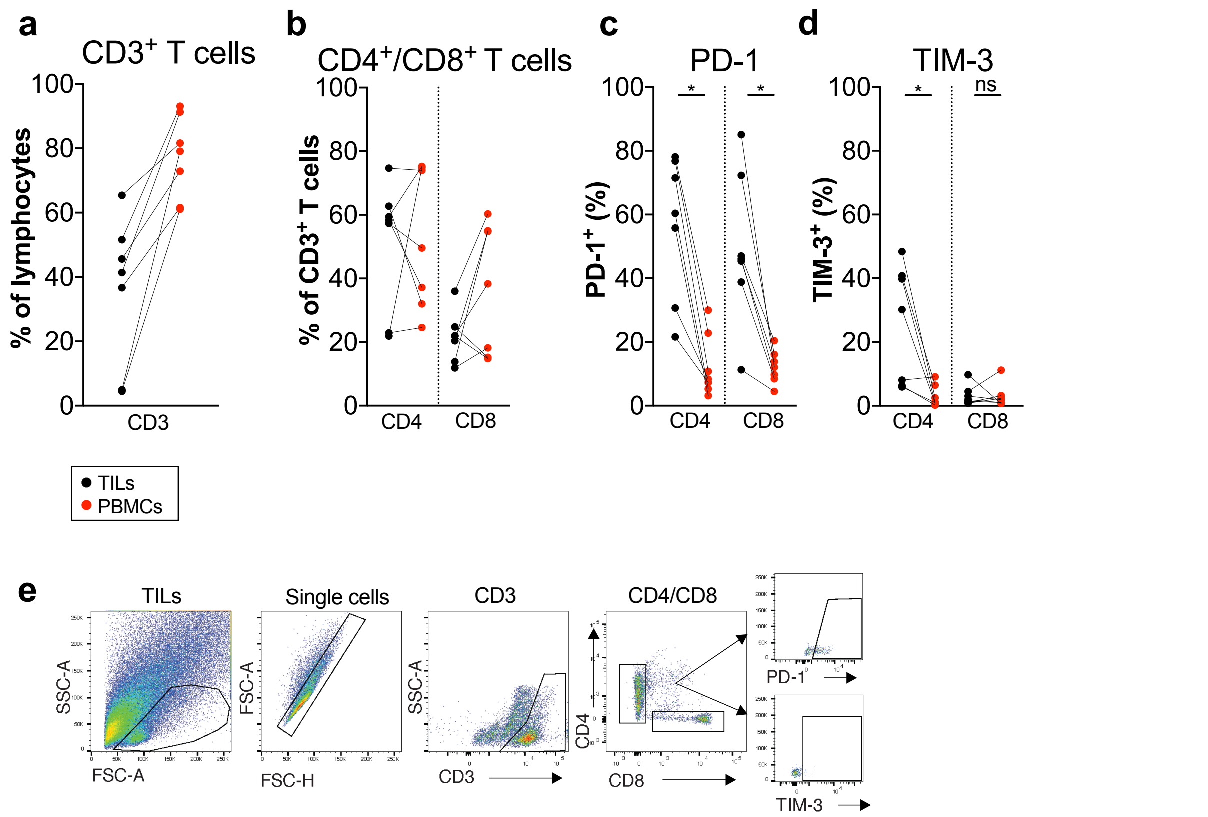

Supplementary Figure S3. Characterization of tumor infiltrating lymphocytes (TILs) and PBMCs. (a-d) Comparison between paired samples of TILs and PBMCs in unstimulated conditions. Frequencies of **(a)** $CD3^+$ T cells within the lymphocyte gate and **(b)** $CD4^+$ and $CD8^+$ T cells out of $CD3^+$ T cells. Expression of **(c)** PD1 and **(d)** TIM-3 on $CD4^+$ and $CD8^+$ T cells. **(e)** Representative flow cytometry plots on TILs showing the gating strategy to identify $CD3^+$ T cells, $CD4^+$ and $CD8^+$ T cells as well as $PD\text{-}1^+$ and $TIM\text{-}3^+$ T cells. **(a-d)** Lines between dots indicate paired samples. Wilcoxon matched-pairs singed rank test was used to detect statistically significant differences $*P<0.05$.

Supplementary Table S1

Gene	Forward Primer (5´to 3´)	Reverse Primer (5´to 3´)
VDR	ACC TGG TCA GTT ACA GCA TCC	TGG TGA AGG ACT CAT TGG AGC
CYP24A	CTC AGC AGC CTA GTG CAG ATT	ACT GTT TGC TGT CGT TTC CAC
CAMP	TCC TCG GAT GCT AAC CTCT	TGA CTG CTG TGT CGT CCT
ACTA2	CGA TGC TCC CAG GGC TGT TT	TTC GTC ACC CAC GTA GCT GTC TTT

Table S1. Forward and Reverse primers for human *VDR*, *CYP24A*, *CAMP* and *ACTA2* genes.

Supplementary Table S2

**Pre-mix solution**		**Acellular collagen layer**		**Cellular collagen layer**	
**Reagents**	**Vol. (μl)**	**Reagents**	**Vol. (μl)**	**Reagents**	**Vol. (μl)**
**5x DMEM (pH 7.2)**	395	Pre-mix solution	50	Collagen I	**400**
**$NaHCO_3$ (71.2mg/ml)**	120	Complete DMEM	22	Pre-mix solution	**123**
**L-glutamine**	39	Collagen I	137	Cell suspension	**65**
**FBS**	440			Complete DMEM	**65**
**PEST**	5				

Table S2. Volume of reagents used to set up the collagen gels. Volumes are calculated for one well of a 24 well-plate

Supplementary Table S3

Markers	Fluorochrome	Clone	Company	Catalogue number	Staining
**CAFs markers**					
**PD-L2**	APC	MIH18	BD	557926	EC
**Podoplanin**	PE-Cy7	NC-08	BioLegend	337007	EC
**PD-L1**	PE	29E.2A3	BioLegend	329706	EC
**αSMA**	A488	1A4	Abcam	Ab184675	IC
**Ki-67**	AF488	B56	BD	561165	IC
**Live/Dead viability**	APC-Cy7	-	BD	565388	
**T cells markers**					
**CD3**	PE-Cy-7	UCHT1	BD	563423	EC
**CD3**	V450	UCHT1	BD	560365	EC
**CD4**	A700	RPA-T4	BD	557922	EC
**CD4**	V500	RPA-T4	BD	560768	EC
**CD8**	APC-Cy7	SK1	BD	557834	EC
**PD-1**	BV421	EH12.1	BD	562516	EC
**HLA-DR**	V500	G46-6	BD	561223	EC
**TIM-3**	APC	F38-2E2	Miltenyi	130120700	EC
**Ki-67**	AF488	B56	BD	561165	IC
**IFN-γ**	PE-Cy7	B27	BD	557643	IC
**Granzyme B**	PE	GB11	BD	561142	IC
**Perforin**	PECF594	δG9	BD	563763	IC
**-**	7AAD	-	BD	559928	

Table S3. List of antibodies used for flow cytometry analysis. Abbreviations; Markers; **PD-L** Programmed death-ligand **CD**, Cluster of differentiation, I, **αSMA**, Alpha smooth muscle actin **HLA-DR**, Human leukocyte antigen-antigen D Related, **TIM-3**, T-cell immunoglobulin and mucin-domain containing-3, **LAG-3**, Lymphocyte activation gene, **IFN-γ**, Interferon gamma. Fluorochromes; **APC**, Allophycocyanine. **BV421**, Brilliant violet 421, **A647**, Alexa Fluor 647, **PE**, Phycoerythrin, **A488**, Alexa Fluor 488, **Pe-Cy7**, Phycoerythrin-cyanine 7, **V450**, Violet 450, **A700**, Alexa Fluor 700, **V500**, Violet 500, **APC-Cy7**, Allophycocyanine-indo tricarbocyanine, **FITC**, Fluoresceinisothiocyanate,, **7-AAD**, 7-Aminoactinomycin D. Companies; **BD**, BD Biosciences (Franklin Lakes, NJ, USA), **BioLegend**, Biolegend (San Diego, CA, USA), **Abcam,** Abcam (Cambridge,UK), **Miltenyi**, Miltenyi Biotec (Bergisch Gladbach, Germany). Stainings; **EC**, Extracellular, **IC**, Intracellular.
